# Measuring forgetting: A critical review of accelerated long-term forgetting studies

**DOI:** 10.1016/j.cortex.2014.02.001

**Published:** 2014-05

**Authors:** Gemma. Elliott, Claire L. Isaac, Nils Muhlert

**Affiliations:** a Clinical Neuropsychology Services, Royal Hallamshire Hospital, Sheffield, UK; b Department of Psychology, University of Sheffield, Sheffield, UK; c Neuropsychology Department, Lincolnshire Partnership NHS Foundation Trust, Lincoln, UK; d Cognitive Neuroscience, School of Psychology, Cardiff University, Cardiff, UK

**Keywords:** Accelerated long-term forgetting, Forgetting rates, Epilepsy, Methodology

## Abstract

Accelerated long-term forgetting (ALF) refers to abnormal forgetting over hours to weeks despite normal acquisition or initial consolidation. Since standardised assessments of memory typically only test at delays of up to 40-minutes, ALF may go undetected in clinical practice. The memory difficulties associated with ALF can however cause considerable distress to patients. It is important therefore that clinicians are aware that ALF may represent a distinct phenomenon that will require additional and careful assessment to aid patients' understanding of the condition and assist in developing strategies to address its effects. At the same time, ALF may also provide insight into long-term memory processes. Studies of ALF in patients with epilepsy have so far demonstrated mixed results, which may reflect differences in methodology. This review explores the methodological issues that can affect forgetting, such as the effects of age, general cognitive function, test sensitivity and initial learning. It then evaluates the extent to which existing studies have considered these key issues. We outline the points to consider when designing ALF studies that can be used to help improve their validity. These issues can also help to explain some of the mixed findings in studies of ALF and inform the design of standardised tests for assessing ALF in clinical practice.

## 1 Introduction

Accelerated long-term forgetting (ALF) refers to the phenomenon whereby memories are encoded and retained normally over delays of up to 30-min, but are then forgotten at an abnormally rapid rate over delays of days to weeks thereafter. The phenomenon was first described in a case study by De Renzi and Lucchelli (1993), and greater forgetting over days in people with epilepsy was first reported by Martin et al. (1991). Since then several further case studies have been published (Butler & Zeman, 2008a; Cronel-Ohayon et al., 2006; Holdstock, Mayes, Isaac, Gong, & Roberts, 2002; Jansari, Davis, McGibbon, Firminger, & Kapur, 2010; Kapur et al., 1997, 1996; Kemp, Illman, Moulin, & Baddeley, 2012; Lucchelli & Spinnler, 1998; Mayes et al., 2003; O'Connor, Sieggreen, Ahern, Schomer, & Mesulam, 1997). Whilst these cases of ALF are associated with a range of aetiologies, the fact that the majority experienced temporal lobe epilepsy (TLE) resulted in a focus on group studies of people with TLE. The phenomenon was originally labelled “long-term amnesia” (Kapur et al., 1997, 1996). However, the term accelerated long-term forgetting was introduced by Blake, Wroe, Breen, and McCarthy (2000) and has subsequently become the most widely used label.

Abnormal forgetting has often been attributed to a failure of memory consolidation (e.g., Isaac & Mayes, 1999a). This is the hypothetical process in which memories become stabilised within long-term storage, through processes of both synaptic and systems level changes (McGaugh, 2000), allowing later retrieval. Whilst consolidation may continue for weeks, months or even years (Squire & Alvarez, 1995) it is generally assumed that its efficacy can be evaluated after relatively short delays, explaining the use of 30-min delays in standardised memory tests. The neurobiological underpinnings of ALF are poorly understood but may benefit from considering theories of long-term memory (LTM). The main theory of consolidation, the Standard Model (Alvarez & Squire, 1994; Squire, 1992; Squire & Alvarez, 1995; Squire, Cohen, & Nadel, 1984), proposes that the Medial Temporal Lobe (MTL) is involved in the initial stages of consolidation but that, over time, memories are reorganised so as to become supported by the neocortex and, eventually, independent of the MTL. Mayes et al. (2003) distinguish ‘fast’ from ‘slow’ LTM consolidation processes. The ‘fast’ consolidation process involves interactions between a number of cortical systems which is thought to be mediated by MTL structures, such as the hippocampus. If ALF reflects a failure of this consolidation system then this could result from subtle MTL damage or abnormal activity, in which functional disconnection between hippocampal and cortical systems prevents memories from becoming established. ALF would therefore represent a mild form of amnesic syndrome. In contrast, the ‘slow’ consolidation process is thought to depend on a stable environment in the temporal neocortex, allowing for repeated and synchronous activation of hippocampal–neocortical connections. In this case, ALF may result from failed slow transfer of information into neocortical storage sites resulting either from structural neuropathology preventing establishment of memories or from disrupted transfer due to epileptiform activity (e.g., Kapur et al., 1997). An alternative theory, the Multiple Trace Theory (Nadel & Moscovitch, 1997), proposes that the MTL is always involved in the stabilisation and retrieval of memories. In this model each reactivation of a memory produces a new trace within the MTL and neocortical regions. Forgetting occurs when memories are not re-activated and so do not benefit from the formation of multiple traces in the MTL and neocortex, or when these processes of stabilisation are compromised (Nadel & Moscovitch, 1997).

Both theories of LTM predict that structural damage and/or seizure activity may disrupt consolidation/stabilisation processes (Mayes et al., 2003). Improved definition of the nature and underlying causes of abnormal forgetting may therefore have important implications for theories of normal memory functioning. To date however, the evidence for ALF in TLE has been mixed (Bell & Giovagnoli, 2007; Butler & Zeman, 2008b; Fitzgerald, Mohamed, Ricci, Thayer, & Miller, 2013), with ALF reported in some TLE studies (Blake et al., 2000; Butler et al., 2007; Mameniskiene, Jatuzis, Kaubrys, & Budrys, 2006; Manes, Graham, Zeman, de Lujan Calcagno, & Hodges, 2005; Martin et al., 1991; Muhlert et al., 2011; Muhlert, Milton, Butler, Kapur, & Zeman, 2010; Tramoni et al., 2011) but not others (Bell, 2006; Bell, Fine, Dow, Seidenberg, & Hermann, 2005; Giovagnoli, Casazza, & Avanzini, 1995). Another issue for studies of ALF, is that patients may show accelerated forgetting over immediate and short delays (e.g., 30 min) as well as very-long delays. In these cases, it is necessary to use procedures which match participants for initial learning and immediate recall.

One serious challenge for assessing ALF routinely is that standardised tests of memory do not assess performance at delays greater than 40 min. As ALF, by definition, occurs beyond this time point, some patients' memory impairment may go undetected. In the absence of standardised tests, researchers have created their own materials and procedures for assessing forgetting over extended delays. The mixed findings in studies of ALF could therefore be explained by differences in methodological approaches and the significant difficulties encountered when comparing normal and pathological forgetting. These methodological problems associated with studying ALF are the same as those encountered when studying any form of forgetting. Considering methodological issues from the wider literature on forgetting (e.g., Isaac & Mayes, 1999a) may therefore help to inform this debate.

This review aims to evaluate methodological problems within forgetting research in general and ALF in particular. In contrast to previous reviews of ALF (Bell & Giovagnoli, 2007; Butler & Zeman, 2008b; Fitzgerald, Mohamed, et al., 2013), this review primarily focusses on the impact of methodology on forgetting rates, rather than the clinical features of patients who show ALF. Revisiting this literature is timely when many researchers are developing new assessments and procedures to study ALF. In Part I, the literature addressing methodological issues in the assessment of forgetting rates is summarised. Rather than trying to resolve the complex theoretical and mathematical debates, this review aims to summarise the different opinions on studying forgetting, evaluate their implications and provide a reference point for issues that should be tackled when assessing ALF. In Part II, we review existing case reports and group studies of ALF with emphasis on experimental design. We evaluate the extent to which key methodological issues have been addressed in each study and describe findings that take into account these quality-related issues.

### 1.1 Search strategy

The initial search strategy is summarised in Table 1 (searches resulting in zero matches are not shown). Broad search terms were used for Part I to avoid biasing the selection of methodological issues. Searches were limited to peer-reviewed, human studies for which the full text was available in English.

Following initial searches, titles and available abstracts were examined for relevance and reference lists were trawled to identify reports which were not indexed. Trawling references proved to be the source of many articles identified for Part I, since their publication dates preceded indexing. Only papers considering methodological factors that could be controlled for in studies of ALF were included. This process resulted in a total of 22 articles being identified as relevant to Part I and 33 articles relevant to Part II.

## 2 Part I: methodological issues in assessing forgetting rates

Three key methodological issues relating to the comparison of forgetting rates were identified: first, selection of appropriate control participants, second, selection of appropriate test material and procedures and third, the degree of initial learning and rate of forgetting.

### 2.1 Selection of control participants

Given that there are no standardised tests (with normative data) for assessing ALF, researchers have had to use control groups to document ‘normal’ patterns of forgetting. It is widely accepted that patient and control groups should be as similar as possible but the variables used to match groups have been debated.

#### 2.1.1 General cognitive functioning/educational background

In healthy people, memory and intellectual function are known to be positively correlated (Mayes, 1986). This means that the extent of pure memory impairment can only be assessed when taking into account that person's intellectual function. Whether *forgetting* is also related to IQ is less clear. There are theoretical reasons why this may be the case. For example, people with higher IQ are likely to create increased numbers of associations and use more efficient organisation of to-be-remembered material, which could attenuate forgetting. However, one of the few studies to assess this relationship failed to show a significant correlation between IQ and forgetting over a 20-min delay (Kopelman & Stanhope, 1997). More systematic study of the effects of IQ on forgetting is needed but until then we recommend matching groups for IQ. This should not necessarily require the full Wechsler Adult Intelligence Scale (WAIS) battery of tests, and has often been done using a limited number of subtests or an abbreviated intelligence scale (e.g., Muhlert et al., 2010). Alternatives for matching general function are to use educational background or measures of premorbid IQ (e.g., tests of reading ability like the National Adult Reading Test – NART). However it has been noted that the difference between estimated premorbid IQ (as measured with reading tests) and current IQ (as measured with WAIS tests) increases with the duration of epilepsy and that patients with long epilepsy durations show greater differences between premorbid and current IQs than those with short epilepsy duration (Jokeit & Ebner, 2002). Similarly, education level will not take into account decline in function linked to epilepsy or other conditions with adult onset.

#### 2.1.2 Age

The existence of an age-related increase in forgetting rates has been heavily debated. Early studies comparing younger and older adults reported that older adults showed greater forgetting on visuo-spatial recognition tests over immediate or 12–24-sec delays (Lehman & Mellinger, 1986; Poon & Fozard, 1980) but little difference in forgetting over delays ranging from 2 min to 24 h (Wickelgren, 1975). However these studies did not attempt to match groups for initial learning. Later studies that did reported accelerated forgetting in older adults on visuo-spatial recognition tests (Huppert & Kopelman, 1989; Park, Puglisi, & Smith, 1986) and on verbal recall tasks (Giambra & Arenberg, 1993).

Age-related increases in forgetting rate were later linked to the type of material to be remembered. Park et al. (1986) found that older adults showed greater forgetting on recognition of complex visual scenes over a 4-week delay than younger adults. However, the same target scenes and distractor scenes were presented at both immediate and 4-week delayed testing points. This meant that successful recognition at the 4-week delay required subjects to identify whether the items were the original stimuli (target) or were the distractor items from the immediate recognition trial (i.e., foils). This difficulty with remembering when information had been seen (called ‘source memory’) was later found to be the primary problem for older adults (Craik, Morris, Morris, & Loewen, 1990; McIntyre & Craik, 1987; Simons, Dodson, Bell, & Schacter, 2004) and was associated with performance on tests of frontal lobe function (Craik et al., 1990).

More recent studies which avoid repetition of distractor stimuli have however shown subtle accelerated forgetting in older adults (Davis et al., 2003; MacDonald, Stigsdotter-Neely, Derwinger, & Bäckman, 2006). Davis et al. (2003) compared verbal recall and recognition performance in four age groups (30–45 years, 46–60 years, 61–75 years, and 76–90 years) on a verbal recall and recognition task after 20-min and 1-day delays. The two eldest age groups recalled fewer words at both delays. In addition, even after selecting only those participants who were matched for initial acquisition, the oldest group still demonstrated accelerated forgetting. Similar findings were observed in a study in which younger and older adults were taught four-digit numbers to perfection then tested for retention after 30-min, 24 h, 7 weeks and 8 months (MacDonald et al., 2006). Older age predicted accelerated forgetting, particularly within the first 24 h. Last, a recent study found that older adults (aged 65–75 years) showed similar forgetting of a list of word-pairs to younger adults (18–30 years) over a 30-min delay but greater forgetting over a 7-day delay, although ceiling effects on this test may have masked early forgetting (Mary, Schreiner, & Peigneux, 2013). In summary, despite generally mixed evidence for an effect of age on forgetting, the weight of evidence currently suggests that some increased forgetting occurs with increasing age. This suggests it is advisable to match groups for age.

### 2.2 Test materials and procedures

Six issues relating to test materials and procedures were identified: material specificity, assessment procedures (e.g., free recall, cued recall, recognition), ceiling and floor effects, matching initial learning, rehearsal effects and influence of short-term memory (STM). We consider each issue and its relevance to studies of ALF.

#### 2.2.1 Material specificity

Many studies have suggested the presence of a material-specific difference in memory functions of the left and right-temporal lobes. These differences often emerge in patients who have undergone temporal lobectomy for the relief of intractable epilepsy. For instance, resection of the left MTL has been fairly consistently associated with impairments in verbal memory (e.g., Kimura, 1963). Resection of the right MTL has been linked to impaired visuo-spatial memory although this relationship is generally less consistent than that between verbal memory and the left MTL (Lee, Yip, & Jones-Gotman, 2002). Material-specific memory deficits have also been reported in patients with TLE who have not undergone surgery. In these studies, patients with left TLE show impaired verbal memory (Delaney, Rosen, Mattson, & Novelly, 1980; Mungas, Ehlers, Walton, & McCutchen, 1985) but, as with post-surgery patients, the association between right TLE and impaired non-verbal memory has proved elusive (Barr, 1997). A recent study highlighted the role of both temporal lobes in visuo-spatial tasks in patients with TLE (Glikmann-Johnston et al., 2008). Furthermore, reviews point out that the generally weak association between right MTL integrity and visuo-spatial memory presents a challenge to the material-specificity model (Baxendale & Thompson, 2010; Saling, 2009). Instead, visuo-spatial memories may rely on a dynamic bilateral interaction between MTL structures, as suggested by Glikmann-Johnston et al. (2008).

Many ALF studies have assessed both verbal and visuo-spatial memory (Bergin, Thompson, Fish, & Shorvon, 1995; Butler et al., 2007; Cronel-Ohayon et al., 2006; Davidson, Dorris, O'Regan, & Zuberi, 2007; De Renzi & Lucchelli, 1993; Helmstaedter, Hauff, & Elger, 1998; Kapur et al., 1997; Lucchelli & Spinnler, 1998; Mameniskiene et al., 2006; Manes et al., 2005; Mayes et al., 2003; Muhlert et al., 2011). In principle, this allows assessment of whether particular forms of information are forgotten at different rates in people with epilepsy, and improves the generalizability of findings (Hart & O'Shanick, 1993). Assessing forgetting rates for different types of material also helps assess whether rapid forgetting reflects a general memory consolidation/stabilisation deficiency or deficits in information processing or memory for particular types of information.

In TLE studies, three showed ALF on verbal but not visuo-spatial material (Davidson et al., 2007; Lucchelli & Spinnler, 1998; Manes et al., 2005) and one showed ALF for a verbal test in left TLE but not right TLE patients (Blake et al., 2000). However, of the studies which did not find ALF on visuo-spatial memory tests, one showed floor effects in both patients and controls (Manes et al., 2005) and a second believed the negative finding to relate to “the reduced ability [of the test] to discriminate differences in recall ability” (p. 398, Davidson et al., 2007), leaving a single case study in which a patient showed ALF for a story but not a complex figure (Lucchelli & Spinnler, 1998). This provides little evidence in support of material-specific ALF. If verbal and visuo-spatial memory tests are used to study ALF, researchers should consider whether differences in performance between tests relate to differences in difficulty and sensitivity before material specificity in forgetting.

Butler and Zeman (2008b) found existing data to be inconclusive regarding whether laterality of seizure focus leads to material-specific forms of ALF. Until such evidence exists, it is advisable to assess both verbal and visuo-spatial material when studying ALF.

#### 2.2.2 Assessment procedure

Memory studies typically use free recall, cued recall and/or recognition procedures to assess forgetting. In free recall paradigms participants are asked to think back to an episode and retrieve memories in the absence of more specific cueing. In cued recall participants are given a specific cue to aid memory retrieval. Recognition tests require participants to correctly remember something they have previously encountered when it is presented again. Recognition may be assessed using a forced-choice procedure (where subjects view two or more items simultaneously and judge which one they have seen before) or yes/no procedures (where subjects view a single item and judge whether or not it has been presented previously). Isaac and Mayes (1999a) found greater forgetting on tests of recall than recognition in patients with amnesia. However many earlier studies of forgetting rates (e.g., Freed & Corkin, 1988; Huppert & Piercy, 1978) focused only on recognition memory. Given evidence that recognition and recall memory may be differentially affected in amnesia, Isaac and Mayes (1999a) argued for the importance of examining both. Some evidence suggests that ALF affects both recall and recognition, but the findings are inconsistent (Butler & Zeman, 2008b). Examining both recall and recognition in studies of ALF may provide further insight into the processes that are affected.

Even within a recognition paradigm, differences in test procedure may be important. For example, Freed and Corkin (1988) compared the performance of patient H.M. on a forced-choice recognition procedure, a yes–no recognition procedure, and a yes–no (new) procedure (where subjects judge whether or not an image is new, focussing on aspects of novelty). Different recognition procedures yielded discrepant results with the least variability seen in the forced-choice procedure. It was unclear why this discrepancy arose but one possibility is that differences in difficulty on the different tests affected findings. This demonstrates the importance of piloting test material to ensure such confounds have minimal impact on results.

#### 2.2.3 Ceiling and floor effects

Ceiling effects arise when a test is not challenging enough for high functioning individuals, who subsequently achieve the maximum score. In contrast, floor effects arise due to task difficulty causing performance to be at the lowest point. Ceiling and floor effects are problematic because in both cases forgetting rates may be underestimated, obfuscating the true group mean. Consequently, the measured statistical variance will be below its true level, reducing the sensitivity of group analyses. Questions have arisen about whether it is preferable to avoid analysing data that appear to approach floor (e.g., Slamecka & McElree, 1983) or to presume that forgetting may still occur (despite not being detectable by the dependent measure) and include the data.

The importance of this issue was highlighted by Isaac and Mayes (1999a), who noted that unless tasks are carefully designed and piloted, participants with memory disorders are at risk of performing at floor levels and control participants at ceiling levels. In ALF studies, ceiling effects at short delays may lead to underestimation of forgetting rates in healthy controls. Systematic piloting and adjustment of stimuli difficulty levels and assessment procedures can help to minimise ceiling and floor effects in ALF studies. This may involve manipulating the length of stimulus presentation, the number of presentations of stimuli, or the interval lengths between testing sessions. These possibilities will be considered in relation to matching levels of performance at the shortest delay.

### 2.3 Matching initial learning

It has been argued that scaling problems (discussed in section 2.6) can be eliminated by matching initial learning (Huppert & Piercy, 1978) across groups of participants. Shuell and Keppel (1970) outlined several matching procedures: administering different numbers of exposure trials, using different lengths of stimuli lists, or employing study intervals of different durations. Although such procedures may successfully equate initial performance, little is known about the consequences of these manipulations on forgetting. Potential matching procedures will now be considered in more detail.

#### 2.3.1 Extended exposure times

Shuell and Keppel (1970) equated learning of word-lists in healthy, student participants using different presentation rates, of 1 or 5 sec. To ascertain which participants should receive the longer presentation rate, participants first had to complete a pre-test which involved remembering a list of words. Their performance was then ranked; those who recalled more words were classified as fast learners (and assigned the shorter presentation rate) and those who recalled fewer words were classified as slow learners (and assigned the longer presentation rate). When retention was tested after 24 and 48 h, slow and fast learners showed similar rates of forgetting.

Huppert and Piercy (1978) used this matching procedure to examine forgetting in patients with organic amnesia. In their study, people with amnesia (*n* = 7) and healthy controls (*n* = 6) were matched for picture recognition after a 10-min interval. Initial learning was matched by presenting each picture for 4 or 8 sec to amnesic participants but for only 1 sec to controls. When tested again 1 day and 7 days later, yes–no recognition performance had declined at similar rates in both groups. This was interpreted as evidence for an initial learning deficit amongst amnesic patients, without concomitant increases in forgetting rates. Learning deficits were therefore rectified by increased exposure time at presentation.

It was later pointed out that Huppert and Piercy's method may have biased against finding accelerated forgetting (Mayes, 1986). Since amnesic participants receive longer exposure to the test stimuli and the delay is timed from the end of the presentation phase, the mean item-to-test delay period is longer for patients than controls. Memory generally decays at an exponential rate, with the majority of forgetting occurring soon after learning. In this paradigm, it is possible that more forgetting has occurred in patients prior to the first recall attempt, so they show less forgetting to later time points than controls. On this basis, Mayes (1986) advocated matching the mean item-to-test delay by calculating the necessary exposure time for the most impaired participant and then ensuring all participants have the same delay between item presentations. For example if poor-learners require 3 sec of exposure, then good-learners could be matched through 1 sec exposure of the stimuli, followed by 2 sec of blank screen. In this case, each trial for each participant lasts a total of 3 sec.

#### 2.3.2 Multiple presentation procedure

Isaac and Mayes (1999a) adopted a multiple presentation procedure as an alternative to the extended exposure method. This primarily allowed use of a story, which clearly cannot be presented with extended exposure. Instead, multiple presentations of the story were given, for instance healthy controls were given one presentation of the story, whereas the memory impaired patients would receive two or three presentations, depending on their level of memory impairment (defined on the basis of performance on standardised memory tests). This matched the group's initial performance whilst maintaining a consistent delay between the final presentation of the stimuli and test.

#### 2.3.3 Learning to criterion

**Fig. 1:**
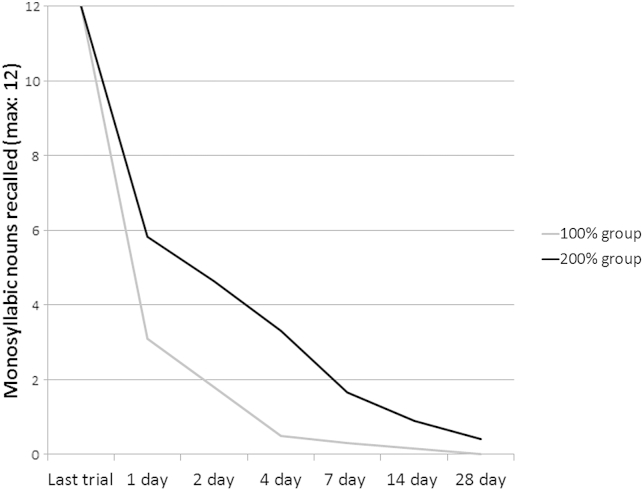
Overlearning of stimuli affects forgetting rates (replicated from Krueger et al., 1929). Half of the participants learnt a list of monosyllabic words to 100% (grey line), the other half learnt to 100% then had the same number of learning trials again (black line). Forgetting rates were decreased in the latter, overlearning, group.

Learning to criterion involves repeatedly presenting material until a criterion (e.g., 100% accuracy on two successive trials) is reached. Bell (2006) argued that this method of matching learning poses the risk of the material being over-learnt; leading to the possibility that early forgetting is masked by ceiling effects. Overlearning is the continued learning of stimuli beyond the criterion of one perfect trial (Krueger, 1929). In his early study of overlearning, Krueger (1929) gave participants either just enough trials to recall a word-list flawlessly, or twice this number of trials (i.e., 100% overlearning; see Fig. 1). When tested after 1-day and 27-day delays, participants in the overlearning condition forgot fewer words than those in the normal learning condition. This reduction of forgetting rates caused by overlearning has been identified across a range of studies but evidence suggests that it is short-lived (Driskell, Willis, & Copper, 1992) and may have greatest effect on forgetting over the first 24 h after learning with less effect over delays of 2–28 days (Driskell et al., 1992). It follows that forgetting studies which use paradigms prone to overlearning may underestimate forgetting over delays up to 24 h. Given that criterion levels are often set at a level which exceeds perfect recall on one trial, overlearning is indeed a risk inherent to this approach. In these cases, forgetting over long delays may also have been apparent over shorter, 30-min delays, yet this was obscured by overlearning. A simple alternative is to set the criterion to a level lower than 100%. A recent study used a criterion of 80% on a word-list, which matched groups without ceiling effects (Muhlert et al., 2010).

A viable alternative may be the selective reminding procedure (Buschke, 1973; Buschke & Fuld, 1974) whereby only non-remembered items are presented again at further learning trials. However, the standard administration of this method also requires that participants recall all items on two consecutive learning trials, necessitating ceiling effects. Limiting further learning trials to sub-ceiling thresholds may more adequately avoid overlearning and subsequent confounds.

In summary, matching initial learning between groups is important to avoid biasing estimates of forgetting. There are a number of different methods for equating initial learning, which are suited to different material, such as extended exposures for lists of stimuli and multiple presentations for stories. Regardless of the chosen procedure, researchers should be mindful of the potential implications in the interpretation of their results.

### 2.4 Rehearsal effects

Rehearsal is the act of repeatedly practicing information to be remembered, which is known to be beneficial for LTM. Since rehearsal effects have not been systematically examined with respect to ALF, to avoid confounding results, the potential for rehearsal during delays should be eliminated where possible (Butler & Zeman, 2008b). Not forewarning participants about later requests for recall is one means of addressing this issue. However, if participants are aware of the nature of the study or if it is a repeat assessment within clinic, they may predict that they will be asked about the information again. Another option is to purposefully select stimuli which are difficult to rehearse. For instance some researchers have used a large number of complex visual scenes (Kemp et al., 2012; Muhlert et al., 2011). It is unclear how much participants will rehearse stimuli, and further information is needed to understand this, however an important point to consider is whether to use different stimuli when patient and control participants are related or close friends. Where this is not possible it can be useful to explicitly request they do not discuss their experiences of the memory test.

A related issue is the potential effects of repeated recall. Jansari et al. (2010) assessed the effect of frequent recall on subsequent memory performance in a TLE case study. Their patient learnt 10 separate stories during the presentation phase. Recall and recognition of two stories were assessed at five time points (30-min, 1 day, 1 week, 2 weeks and 4 weeks). The remaining eight stories were tested in pairs using recall and recognition at one time point only (stories 3&4 tested at 1 day, stories 5&6 at 1 week, stories 7&8 at 2 weeks, and stories 9&10 at 4 weeks). Comparing free recall and recognition data across stories, the results suggested that repeated recall had a protective effect against forgetting, without which story recall fell to floor levels within 2 weeks. This study illustrates that repeated recall (without re-presentation of stimuli) may help counteract the effects of ALF. Other studies have attempted to avoid the problem of repeated retrieval by presenting different stimuli at each delay (Evans, Elliott, Reynders, & Isaac, 2013; Muhlert et al., 2010), and by using large stimuli sets (Evans et al., 2013). This can help to avoid confounds created by repeated retrieval.

### 2.5 STM influence

Studies which match performance between groups at an immediate delay may be confounded by the risk that performance is partially based on STM. STM refers to the capacity to hold a limited amount of information in mind for a period of seconds or until distraction (Baddeley, 2012). In healthy participants, recall of the last few items of a list (i.e., the recency effect) is diminished when participants are asked to count numbers after learning but before recall (Glanzer & Cunitz, 1966). Imposing this *distractor task* was argued to prevent rehearsal of items, removing the support of STM from retrieval. In many patients with LTM problems, STM is relatively unaffected. Since immediate recall can benefit from STM whereas delayed recall cannot, this can lead to spurious findings of accelerated forgetting. Ensuring that information is retrieved from LTM at both time points rules out the possibility that poor delayed recall represents a disruption in the transfer process between STM and LTM as opposed to forgetting from LTM alone. Use of a 15-sec distractor task (Isaac & Mayes, 1999a, 1999b; Muhlert et al., 2011) prior to immediate recall has been used to ensure that immediate retrieval is not boosted by STM processes.

With regard to investigating ALF, best practice would be to test participants following a filled delay of at least 10 sec, as contributions from STM will have largely decayed by this time (Cowan, 1993). This allows for more accurate measurement of initial learning and consolidation, lessens the confounding effects of storage in STM, and improves the validity of assessing forgetting from LTM. The inclusion of another test after approximately 30-min then allows for analysis of the forgetting curve in LTM. This procedure will provide evidence that impairments observed at very-long delays (such as days or weeks) signify true ALF rather than memory impairment of the amnesic-type which could be picked up at shorter delays.

To summarise, in developing assessments to study ALF, a combination of verbal and non-verbal material should be used, incorporating tests of recall and recognition. Stimuli should be piloted carefully to establish the type of material and paradigms which induce least variability, have a low risk of floor and ceiling effects and a limited potential for rehearsal. Of further note, procedures should also ensure that immediate recall is based on LTM processes alone.

### 2.6 Degree of initial learning and rate of forgetting

There is considerable debate in the literature regarding the comparison of forgetting rates between groups who may be performing at very different levels. There are two main hypotheses to consider. The first maintains that degree of initial learning does not influence subsequent rates of forgetting (Slamecka, 1985; Slamecka & McElree, 1983) whilst the second argues that forgetting rates cannot be compared unless initial learning is equated (Loftus, 1985a, 1985b).

Slamecka and McElree (1983) argued for the first hypothesis based on forgetting rates of categorized word-lists, paired-associate lists and sentence lists in healthy subjects. Participants were given either one study trial (low degree of learning) or three study trials (high degree of learning) and retention was tested with free recall and cued recall at three intervals (immediate, 1 day and 5 days). Across experiments, the number of study trials affected initial learning levels but had little effect on forgetting rates. Slamecka and McElree concluded that variations in degree of learning are independent of the subsequent course of normal forgetting and argued that equating initial acquisition is not necessary.

Loftus (1985a, 1985b) later argued against this point. He noted that, where immediate performance differs significantly between two groups, comparisons of forgetting can be affected by scaling problems. Loftus presented a model based on the decay of radioactive material. Where two chunks of radioactive material (one large and one small) have the same half-life, there will be a more rapid loss of weight in the larger chunk, than in the smaller. This analogy was then applied to forgetting: groups performing at higher levels have more to forget. A second part to the problem of scaling concerns the level of difficulty of items (Keppel & Wickens, 2004). When a scale is developed and applied to groups with differing abilities, it can differentiate between good performers by using many difficult items (in which case poor performers are clustered at the bottom of the scale), or between poor-learners by using many easy items (in which case good performers are clustered at the top of the scale). When groups are not matched for learning and a loss of *X* number of items occurs, this loss is assumed to have the same meaning at the top and bottom of the scale. Yet a loss of, for example, six difficult items may reflect less forgetting than a loss of six easy items. According to this scaling problem, where different amounts of learning occur, rates of forgetting may be underestimated in groups with lesser degrees of learning. To circumvent this problem, Loftus proposed an alternative method that involves comparing the horizontal distance between forgetting curves over time. This assesses the time taken for two groups to forget *X* amount of items, assuming that, over time, the forgetting curves of the two groups overlap. After analysing previous data using this method, Loftus concluded that a higher degree of original learning leads to a slower rate of forgetting reinforcing the belief that initial learning between groups must be equated.

Whilst definitive conclusions are elusive, an awareness of these debates will assist researchers in making sound methodological decisions. As Wixted (1990) pointed out, the researcher's primary objective is likely to determine the most appropriate method. Despite this, most researchers have continued to assess forgetting rates without any apparent consideration of the methodological issues (Paul, 1994). A simple solution to the problem of scaling is to ensure groups are matched for learning as closely as possible during the presentation phase, yet this does not always occur in practice. Methods for dealing with scaling problems are discussed in Part II.

### 2.7 Analysing forgetting rates

**Fig. 2:**
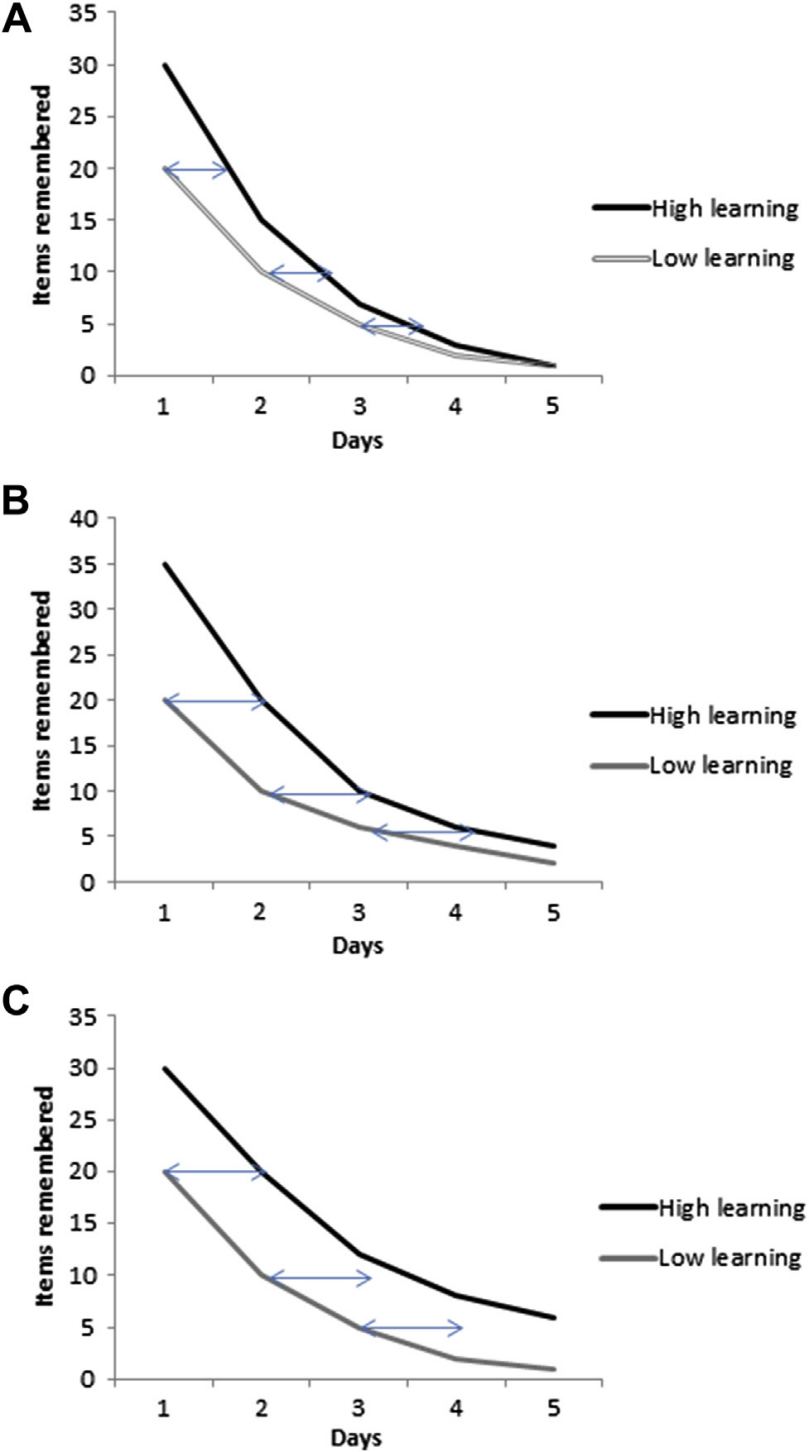
Hypothetical forgetting curves. Loftus (1985a, 1985b) suggested that forgetting curves can be compared by examining their ‘horizontal relation’. The double-ended arrows are the same length for each example. In (A) the high-learning group (black line) shows faster forgetting than the low-learning group (grey line), as shown by the double-ended arrow outgrowing the distance between curves. In (B) the distance remains the same, indicating similar forgetting rates between the two groups, and in (C) the high-learning group shows less forgetting.

Most ALF studies have analysed forgetting using either the number of items forgotten between delays, the group × time interaction term in repeated measures analyses of variance (ANOVAs), which assesses differences in forgetting rates between groups or analysed percent retention scores between delays. If groups are matched for learning, then all three methods should provide reliable results. However where learning differs, using percentage retention could provide unreliable findings. This is illustrated by considering analysing forgetting in terms of number correct or number of errors (A. Baddeley, personal communication). Suppose a high-learning group drops from 80 correct to 50 correct, and a low-learning group from 70 to 40. In terms of percentage loss based on initial score, the low-learners will be seen as forgetting more. However if errors rather than correct items are measured, the errors in the high-learners increase from an average of 20 to an average of 50 (150%), while the low-learners go from 30 to 60 (100%). In this situation it is unclear who is forgetting more. A solution provided by Loftus (1985b) was to examine the ‘horizontal relation’ between forgetting curves (Fig. 2). Where forgetting rates are similar between groups, the horizontal distance (i.e., the time to forget × number of items) between points should remain parallel. This method however introduces another bias: when high-learning and low-learning groups are horizontally aligned, the memories will be older in the high-learning group. In this case horizontally parallel forgetting would mean that older, supposedly stronger, memories are lost in the high-learning group over the same timeframe as younger, supposedly weaker, memories in the low-learning group. Ideally, these issues should be avoided by matching groups for initial performance. Last, analysing performance at individual time points does not assess forgetting itself, so should be avoided. For instance, the difference between the mean scores of two groups could approach significance at time point A, and be significantly different at time point B, which could show some worsening, but does not necessarily indicate significant differences in rates of forgetting.

### 2.8 Part I: summary and recommendations

This review has identified the following key methodological considerations which researchers should take into account when designing ALF experiments. The following recommendations are made based on the previous review:

1. Patient and control groups should be matched, at least for age and intellectual ability.
2. Ideally, both verbal and non-verbal test material should be used.
3. Ideally, forgetting should be measured using both recall and recognition tests.
4. Ceiling and floor effects should be avoided as far as possible.
5. The potential for rehearsal and repeated recall should be avoided as far as possible.
6. The immediate delay period should be long enough to ensure information is stored in LTM and retrieval is not reliant on STM processes.
7. Effort should be made to equate initial learning (whilst avoiding overlearning).

## 3 Part II: do recent studies of ALF in epilepsy meet the recommendations?

Thirty-three studies investigating ALF in epilepsy have been identified. Many of the studies included have already been reviewed elsewhere (Bell & Giovagnoli, 2007; Butler & Zeman, 2008b; Fitzgerald, Mohamed, et al., 2013). However, our specific focus is on methodology and evaluating the extent to which key methodological issues have been considered.

### 3.1 Overview of case reports

Twelve case reports of ALF in epilepsy were identified (Butler, Kapur, Zeman, Weller, & Connelly, 2012; Butler & Zeman, 2008a; Cronel-Ohayon et al., 2006; Holdstock et al., 2002; Jansari et al., 2010; Kapur et al., 1997, 1996; Kemp et al., 2012; Lucchelli & Spinnler, 1998; Mayes et al., 2003; McGibbon et al., 2013; O'Connor et al., 1997). Three pairs of studies report data on the same patients, J.L. (Holdstock et al., 2002; Mayes et al., 2003), R.Y. (Jansari et al., 2010; McGibbon & Jansari, 2013) and P.A. (Butler et al., 2012; Kapur et al., 1997). All cases studied were adults with the exception of Cronel-Ohayon et al. (2006). Demographic data of participants and the main findings of case studies can be viewed in Table 2.

In many case studies, the participant had a history of TLE amidst complex aetiologies, namely closed head injury (Holdstock et al., 2002; Kapur et al., 1996; Mayes et al., 2003), paraneoplastic limbic encephalitis (O'Connor et al., 1997), and late-onset seizures with no clear cause (Butler et al., 2012; Butler & Zeman, 2008a; Jansari et al., 2010; Kapur et al., 1997; Kemp et al., 2012; Lucchelli & Spinnler, 1998). Structural brain imaging was abnormal in all cases with the exception of two patients (Jansari et al., 2010; Lucchelli & Spinnler, 1998). Post-mortem histological analysis of patient P.A. (described in Kapur et al., 1997) later demonstrated neuronal loss and gliosis in both the left and right hippocampus, but little extra-hippocampal damage (Butler et al., 2012). Damage was limited to the temporal lobes in all but the case presented by Holdstock et al. (2002) and Mayes et al. (2003).

### 3.2 Overview of group studies

Nineteen group studies of ALF in adults were identified (Bell, 2006; Bell et al., 2005; Blake et al., 2000; Butler et al., 2009, 2013, 2007; Evans et al., 2013; Fitzgerald, Thayer, Mohamed, & Miller, 2013; Giovagnoli et al., 1995; Helmstaedter et al., 1998; Hoefeijzers, Dewar, Della Sala, Zeman & Butler, 2013; Mameniskiene et al., 2006; Manes et al., 2005; Martin et al., 1991; Muhlert et al., 2011, 2010; Narayanan et al., 2012; Tramoni et al., 2011; Wilkinson et al., 2012), with four analysing ALF data from the same group of patients (Butler et al., 2009, 2013, 2007; Hoefeijzers et al., 2013). Two studies examined ALF in children with idiopathic generalized epilepsy (Davidson et al., 2007; Gascoigne et al., 2012). Demographic data of participants and the main findings of group studies can be viewed in Table 3.

The majority of adult studies sampled TLE patients. Six studies (Butler et al., 2009, 2013, 2007; Hoefeijzers et al., 2013; Manes et al., 2005; Muhlert et al., 2010) report data on patients with Transient Epileptic Amnesia (TEA), a syndrome of epilepsy in which memory problems are particularly common.

All studies identified will now be reviewed for their adherence to the methodological considerations established in Part I. Summaries of the extent to which case studies and group studies met recommendations can be seen in Tables 4 and 5.

### 3.3 Selection of control participants

The recommendation from Part I was that patient and control groups should be matched for age and intellectual ability. All studies with the exception of Martin et al. (1991) successfully matched patients and controls for age. Regarding matching groups for intellectual ability, there is a discrepancy in the way this is achieved. The three methods used for matching are premorbid IQ as measured by the NART or Wechsler Test of Adult Reading (WTAR), number of years in education or current intellectual functioning as measured by WAIS. With neurologically impaired groups, matching intellectual function using current ability is likely to provide the greatest validity. Matching by premorbid ability (as predicted by a reading-derived score or number of years in education) may not take into account any decline from previous ability.

Seven group studies did not match patients and controls for IQ (Bell, 2006; Bell et al., 2005; Gascoigne et al., 2012; Giovagnoli et al., 1995; Helmstaedter et al., 1998; Mameniskiene et al., 2006; Martin et al., 1991). Three of these studies used IQ as a covariate when analysing forgetting (Gascoigne et al., 2012; Helmstaedter et al., 1998; Martin et al., 1991), but it is unclear whether this is a satisfactory resolution to the problem (Adams, Brown, & Grant, 1985). Most studies matched groups on the basis of current intellectual function although some used premorbid IQ (Fitzgerald, Thayer, et al., 2013; Manes et al., 2005; Narayanan et al., 2012). With respect to case studies, only four matched participants for current IQ (Holdstock et al., 2002; Jansari et al., 2010; Kapur et al., 1997; Mayes et al., 2003).

### 3.4 Test materials and procedures

The materials used in ALF studies have varied considerably. Some used standardised tests and added a longer delay whereas others have designed new material. The most commonly adapted existing tests are the Wechsler Memory Scale-Revised (Bell, 2006; Kapur et al., 1997, 1996; Manes et al., 2005; Tramoni et al., 2011), Rey Auditory Verbal Learning Test (Butler et al., 2009, 2013, 2007; Cronel-Ohayon et al., 2006; Helmstaedter et al., 1998; Hoefeijzers et al., 2013; Mameniskiene et al., 2006) and Rey–Osterreith Complex Figure (Cronel-Ohayon et al., 2006; Lucchelli & Spinnler, 1998; Mameniskiene et al., 2006; Mayes et al., 2003; Wilkinson et al., 2012).

Three studies have used ecologically valid stimuli (Helmstaedter et al., 1998; Muhlert et al., 2010; Tramoni et al., 2011). Helmstaedter et al. (1998) devised an assessment of ALF, termed a ‘Memory in Reality Test’, in which participants' memory for the testing session was examined after a 1-week delay. However there was no evidence that the participant could recall this information on the day of the initial testing session, which is problematic for inferring forgetting. In contrast, Tramoni took participants to the cafeteria and later asked them about these events after both short and long delays. Patients with TLE showed normal recall of these details at 1 h relative to healthy controls, but impaired recall at 6 weeks. Muhlert et al. (2010) assessed memory for events captured using an automatic camera on the same day of the event and after 1 day, 1 week and 3 weeks. Patients with TEA showed poorer recall of events and associated details after 24 h. Forgetting of the everyday events correlated with forgetting on a word-list, suggesting the ecological validity of using word-lists to assess ALF.

Ideally, more refined tests should be developed specifically for the assessment of ALF. To aid this, we consider the types of tests which are sensitive to ALF.

#### 3.4.1 Material specificity and assessment procedures

The conclusions drawn in Part I indicated that studies should employ both verbal and non-verbal test materials and evaluate forgetting using a combination of recall and recognition paradigms. This has been met to varying degree in ALF studies.

Of the eleven case reports identified, eight employed verbal and visuo-spatial test material (Butler et al., 2012; Butler & Zeman, 2008a; Cronel-Ohayon et al., 2006; Kapur et al., 1997, 1996; Kemp et al., 2012; Lucchelli & Spinnler, 1998; Mayes et al., 2003), only four of which assessed recall and recognition in both modalities (Butler et al., 2012; Kapur et al., 1997, 1996; Mayes et al., 2003). Of the seventeen group studies, fourteen employed verbal and visuo-spatial material (Bell et al., 2005; Butler et al., 2009, 2013, 2007; Davidson et al., 2007; Evans et al., 2013; Fitzgerald, Thayer, et al., 2013; Helmstaedter et al., 1998; Mameniskiene et al., 2006; Manes et al., 2005; Muhlert et al., 2011; Narayanan et al., 2012; Tramoni et al., 2011; Wilkinson et al., 2012), six of which assessed recall and recognition in both modalities (Butler et al., 2007; Davidson et al., 2007; Evans et al., 2013; Manes et al., 2005; Muhlert et al., 2011; Tramoni et al., 2011). Studies failing to include verbal and non-verbal material are limited by their inability to claim strong evidence for material specificity. For example, in the absence of non-verbal tasks, Blake et al. (2000) could not offer an explanation for subjective reports of memory difficulties in patients with right TLE who performed adequately on verbal tasks. On the other hand, using multiple tests may result in multiple comparison problems. This can be remedied by comparing forgetting across multiple tests using multivariate repeated measures ANOVAs (see Muhlert et al., 2011).

#### 3.4.2 Floor and ceiling effects

The recommendation from Part I was that floor and ceiling effects should be avoided as far as possible. It is not clear from the information published to what extent most studies endeavoured to do this. Floor effects or ceiling effects arose to some extent in all case studies with the exception of Cronel-Ohayon et al. (2006). A common problem is that the performance of patients at long delays is frequently at floor level (at least for some tests). Holdstock et al. (2002) made a concerted effort to ensure tests were sensitive by avoiding floor effects on an item-by-item basis. However, their experimental manipulations were hampered by ceiling effects at 24 h. Holdstock and colleagues acknowledge that this may have concealed forgetting in their patient between 24 h and 3 weeks.

Floor effects were also problematic in group studies by Blake et al. (2000) and Manes et al. (2005). In Blake et al. (2000), five of the left-temporal lobe group and one right-temporal lobe patient scored at floor on story recall after 8 weeks. Manes et al. (2005) found that four patients scored zero on story recall at 6 weeks. In addition, design recall data was not analysed due to all patients and many controls performing at floor levels.

Future studies would benefit from greater consideration of floor and ceiling effects through careful piloting of their test material. This can be achieved by manipulating the length of the long delay, testing at multiple long delay points and varying task difficulty across delays.

#### 3.4.3 Rehearsal effects

Part I demonstrated that the potential for rehearsal should be avoided where possible, however few publications comment on whether this issue was considered. Where rehearsal has been minimised, researchers have not informed participants of later testing sessions (Bell, 2006; Bell et al., 2005; Helmstaedter et al., 1998; Holdstock et al., 2002; Martin et al., 1991; Mayes et al., 2003; Muhlert et al., 2011). However, in order to develop repeatable tests for clinical practice, participants will need to be informed that their memory will be examined again to avoid creating future confounding variables (such as when participants are tested for ALF on multiple occasions, e.g., after starting new treatments or following neurosurgery). An alternative is to explicitly request that participants do not rehearse the material, an approach adopted by Blake et al. (2000), Butler et al. (2007), Davidson et al. (2007), Evans et al. (2013) and Muhlert et al. (2010).

A further issue is the inappropriateness of recruiting friends and family for control groups (Bell, 2006; Bell et al., 2005; Blake et al., 2000; Muhlert et al., 2010). Although family members and friends were asked not to discuss the measure, the likelihood that most people would still be tempted to discuss the process remains. This can be assessed in future studies by explicitly asking participants if they discussed the testing material. Therefore the probability of rehearsal is increased. If there is no alternative, as Blake et al. (2000) and Butler et al. (2007) ensured, care should be taken to ensure that family members are presented with different material.

#### 3.4.4 Delay period

The importance of ensuring that information is stored in LTM prior to an immediate delay test was argued in Part I. The recommendation is that there should be a filled delay of at least 10 sec to eliminate the risk that immediate retrieval is reliant on STM processes. Five studies (Evans et al., 2013; Holdstock et al., 2002; Mayes et al., 2003; Muhlert et al., 2011, 2010) used filled delays and one used an unfilled delay (Wilkinson et al., 2012) to account for this. Of note, studies that used modified versions of existing clinical memory tests are unlikely to have added a filled delay before immediate recall. All studies however did include a 30-min delay which is critical for claiming reliable evidence of ALF.

### 3.5 Matching initial learning

No consensus has been reached regarding whether or not degree of initial learning affects rate of forgetting. This complicates interpretations in studies which chose to accept different acquisition levels and compare the overall shape of forgetting curves over time (Bell, 2006; Bell et al., 2005; Mameniskiene et al., 2006). The conclusion from Part I is that matching initial learning is important to avoid scaling problems when analysing forgetting.

All case study patients achieved comparable immediate recall to controls with the exception of story recall in the case presented by Lucchelli and Spinnler (1998). Largely, this occurred without manipulating presentations, however two studies (Kemp et al., 2012; O'Connor et al., 1997) taught participants to criterion and one (Holdstock et al., 2002) allowed participants greater exposure to the items which would be tested after longer delays. Sixteen group studies (Bell et al., 2005; Blake et al., 2000; Butler et al., 2009, 2013, 2007; Davidson et al., 2007; Evans et al., 2013; Fitzgerald, Thayer, et al., 2013; Gascoigne et al., 2012; Giovagnoli et al., 1995; Hoefeijzers et al., 2013; Martin et al., 1991; Muhlert et al., 2011, 2010; Narayanan et al., 2012; Wilkinson et al., 2012) manipulated experimental procedures in an effort to match initial learning. In most cases this was largely successful, however when performance is at or near ceiling on immediate or short delay trials (e.g., Blake et al., 2000; Butler et al., 2007; Gascoigne et al., 2012), it becomes difficult to judge whether learning was successfully equated. A limitation of studies where initial learning was not matched (Bell, 2006; Bell et al., 2005; Mameniskiene et al., 2006) is that patients' subsequent forgetting rates may have been underestimated as they had less to forget.

Of those studies which attempted to equate initial learning, seven taught participants to criterion (Blake et al., 2000; Butler et al., 2007; Davidson et al., 2007; Gascoigne et al., 2012; Martin et al., 1991; Muhlert et al., 2010; O'Connor et al., 1997), however the potential limitations associated with overlearning were only overtly considered in one (Muhlert et al., 2010). Three studies (Bell et al., 2005; Giovagnoli et al., 1995; Martin et al., 1991) used the selective reminding technique (Buschke, 1973; Buschke & Fuld, 1974) which in part circumvents the issue of overlearning. Three studies applied the multiple presentation procedure (Evans et al., 2013; Muhlert et al., 2011; Wilkinson et al., 2012).

An interesting recent study examined the impact of selecting participants with specific rates of learning. Hoefeijzers et al. (2013) reanalysed list-learning data from Butler et al. (2007), excluding those patients and controls with exceptionally fast or slow learning rates. In addition, only words recalled a set number of times were analysed, ensuring that the groups were matched for both the number of exposures and the number of successful retrievals. This helps to account for both differences in retrieval practice during learning and the level of encoding (assuming that words recalled more often could be encoded at a more ‘deep’, or semantic, level). Despite this precise matching, patients with TEA still demonstrated faster forgetting over 1-week and 3-week delays. This provides further evidence to the robustness of ALF in patients with TEA, suggesting that it cannot be accounted for by an acquisition deficit, and may instead reflect difficulties with memory consolidation/stabilisation.

## 4 Summary and conclusions

This review identified seven methodological issues which are important to take into account when investigating ALF. More specifically, it is recommended that groups are matched for age and intellectual ability, that both verbal and non-verbal tests are used in combination with recall and recognition paradigms and that distractor tasks are used to make it more likely that when retention is tested longer-term memory will be engaged. In addition, experimental manipulations should be made to equate initial learning, avoid ceiling and floor effects and minimise opportunities for rehearsal of test material. Studies of ALF have generally focussed on the clinical features associated with ALF, including the influence of structural damage (Butler et al., 2009, 2012, 2013; Tramoni et al., 2011; Wilkinson et al., 2012), surgery (Evans et al., 2013), seizures (Fitzgerald, Thayer, et al., 2013; Mameniskiene et al., 2006), and other epilepsy-related variables (Fitzgerald, Thayer, et al., 2013), which have influenced their specific design (for a discussion of these findings, see Zeman, Butler et al., 2013). However all have to different extents considered the methodological issues we outlined.

Existing studies investigating ALF in epilepsy were then evaluated to determine whether pertinent methodological issues were considered. On this basis, Mayes et al. (2003) fulfil the greatest number of recommendations. Whilst the patient's verbal recall was at floor at the 3-week delay, ALF was indicated given their recall of the story was within normal limits at 20 sec and 30 min. In this case, a shorter delay of 1 week may have elicited non-floor-level performance. In contrast, the case report by O'Connor et al. (1997) only met the recommendation to match initial learning. In this study, the patients' brother acted as control participant. No formal measures of IQ were taken, although they were considered to have similar educational backgrounds. Furthermore, only verbal recall was assessed making it unclear whether any material-specific deficit existed. Nevertheless, as one of the earliest studies of this unusual pattern of forgetting, O'Connor and colleagues raised pertinent theoretical questions for further investigation.

A significant limitation within group studies has been the difficulty of matching groups for IQ. Unfortunately this is likely to remain a challenge in patient groups who often show low average IQs. Some studies matched for intellectual ability on the basis of reading ability (i.e., premorbid IQ tests) but this may lead to inaccurate matching of neurologically impaired samples (as discussed in the section on general cognitive function). The majority of group studies have employed verbal and visuo-spatial test material, a procedure which should be followed consistently alongside the routine inclusion of both recall and recognition tests. Encouragingly, initial learning was equated and floor and ceiling effects were avoided in many cases, however consideration should be given to the most appropriate means of achieving this. The majority of studies also endeavoured to prevent rehearsal; it is however difficult to ascertain the success of the methods employed. The most reliable option for future studies may be to specifically select stimuli that are difficult to rehearse. Only one group study included a filled delay before immediate recall, a practice that should be adopted in future to ensure forgetting between each delay reflects forgetting from LTM.

This review highlights the need to have appropriate tests for assessing ALF. Ideally, this would involve the creation of a set of standardised clinical ALF tests, all with suitable sensitivity and matched for difficulty, that: (i) use both verbal and non-verbal stimuli, (ii) allow testing with recall and recognition, (iii) have separate matched sets, offering the possibility for repeated testing. Given these tests it would then be possible to assess:

1. Whether forgetting rates vary with general cognitive function or educational background.
2. How much repeated testing, and awareness of the nature of very LTM testing, affect forgetting rates.
3. Whether there are clear physiological or neurobiological correlates of ALF.
4. How ALF relates to psychosocial function.
5. Whether rates of forgetting relate to the difficulty of rehearsing stimuli.

To conclude, existing studies suggest that ALF may be characteristic of patients with TLE. Whilst methodological issues have not always been considered, the demonstration of ALF despite these difficulties suggests the robustness of this particular memory disorder. Future ALF studies would however benefit from improved, comparable methodology. Of most importance is to systematically pilot a range of verbal and non-verbal tests to identify which offer the most reliable measure of ALF. It is also prudent for researchers to bear in mind the clinical importance of investigating ALF and aim to develop repeatable standardised tests which would eventually be suitable for use in clinical practice.

## Figures and Tables

**Table 1:** Summary of initial search.

	Search terms	Database	Matches
*Part I*	“Forgetting rates” OR “rate of forgetting” OR “accelerated long-term forgetting” OR “long-term amnesia” OR “long-term forgetting”	PsychINFO	167
		MEDLINE	122
		Web of Knowledge	1476
	“overlearning” AND “forgetting”	PsychINFO	2
*Part II*	“Accelerated long-term forgetting” AND “epilepsy”	PsychINFO	5
		MEDLINE	28
		Web of Knowledge	13
	“Long-term amnesia” AND “epilepsy”	PsychINFO	3
		MEDLINE	65
		Web of Knowledge	6

**Table 2:** Demographic details and main findings in case studies of ALF.

Authors (year)	ALF evidence? (delay 1st seen)	Sample size		Mean age (SD)		Sex		IQ (SD)		Brain pathology	Seizure lateralization
		Patient	Controls	Patient	Controls	Patient	Controls	Patient	Controls	Patient	Patient
Butler et al. (2012)	Yes (40 days)	1 (P.A.)	4	62	65.3	F	F	124	121.1	Left-HC	L
Butler and Zeman (2008a)	Yes (1 week)	1	24	50	67.7	M	10M 14F	136	120	Left-HC	L
Cronel-Ohayon et al. (2006)	Yes (7 days)	1 (J.E.)	1	18	18	M	M	90	–	L-AMG	L
Holdstock et al. (2002)	Yes (3 weeks)	1 (J.L.)	10	40	41.1 (3.03)	F	F	122	113.5 (8.2)	Bi-TL HC normal	R
Jansari et al. (2010) Experiment 1	Yes (1 day)	1 (R.Y.)	8	63	66.3 (4.9)	M	3M – 5F	118	117.9 (6.29)	None	R
Experiment 2	Yes (1 day)	1 (R.Y.)	6	63	61.8 (5.41)	M	M	118	122 (5.79)		R
Kapur et al. (1996)	Yes (6 weeks)	1 (S.P.)	3	50	46.3	F	F	94	–	Left-TL	–
Kapur et al. (1997)	Yes (40 days)	1 (P.A.)	4	62	65.3	F	F	124	121.1	Left-HC	L
Kemp et al. (2012)	Yes (4 days)	2 (S.K., E.B.)	10 young, 4 old	36, 73	–	M, M	M	92, 124	101.2 young, 127 old	Right-TL, normal	L, –
Lucchelli and Spinnler (1998)	Yes (7 days)	1 (G.B.)	2	65	63.5	M	–	120	–	Mild ventricular asymmetry L > R	L
Mayes et al. (2003)	Yes (3 weeks)	1 (J.L.)	10	41	40.6 (2.7)	F	–	122	116 (10.9)	Bi-TL HC normal	R
McGibbon and Jansari (2013)	Yes (55 min)	1 (R.Y.)	5	68	66.3 (4.9)	M	2M – 3F	116	117.9 (6.29)	None	R
O'Connor et al. (1997)	Yes (24 h)	1 (J.T.)	1	42	40	M	M	127	–	Bi-MTL	Bi

M = male, F = female, – = information not presented, R = right, L = left, Bi = bilateral, TL = temporal lobe, MTL = mesial temporal lobe, HC = hippocampus, AMG = amygdala.

**Table 3:** Demographic details and main findings in group studies of ALF.

Authors (year)	ALF evidence? (delay)	Sample size		Mean age (SD)		Sex		IQ (SD)		Brain pathology	Seizure lateralization
		Patients	Controls	Patients	Controls	Patients	Controls	Patients	Controls	Patients	Patients
Bell et al. (2005)	No (24 h)	42	49	37 (11.4)	37 (11.8)	14M 28F	22M 27F	93.5 (14.2)	104 (12.7)	None	20R 22L
Bell (2006)	No (2 weeks)	25	25	39 (10)	35 (11)	10M 15F	8M 17F	94 (12)	104 (10)	None 6 postop	6R, 11L, 2 Bi 24% uncertain
Blake et al. (2000)	Yes (8 weeks)	21 (14 TLE)	16	33.76 (9.72)	46.25 (14.54)	7M 14F	6M 10F	103.65 (12.72)	101.88 (13.20)	HS 5/14 TLE group	10R 11L
Butler et al. (2007)	Yes (1 week)	24	24	67 (8.7)	67.7 (8.2)	14M 10F	10M 14F	124.3 (10.4)	120 (14.4)	None	–
Butler et al. (2009)	Yes (1 week)	22	20	66.4 (8.8)	67.5 (8.6)	12M 10F	8M 12F	124.7 (10.7)	121.2 (14.9)	<HC volume	–
Butler et al. (2013)	Yes (1 week)	22	20	66.4 (8.8)	67.5 (8.6)	12M 10F	8M 12F	124.7 (10.7)	121.2 (14.9)	<HC, perirhinal volume	
Davidson et al. (2007)	Yes (1 week)	21	21	11.5	11.9	7M 14F	–	99.4 (14.4)	98.5 (11.6)	–	IGE
Evans et al. (2013)	Yes (1 week)	7	25	39.71 (15.8)	38.1 (14.6)	3M 4F	12M 13F	94.0 (8.2)	99.4 (4.7)	5 MTS, 1 left AMG abnormality, 1 right HC volume loss	4R 3L
Fitzgerald, Thayer, et al. (2013)	Yes (24 h)	39	15	32.6–41.8	40.4 (10.9)	–	–	104–108	108.9 (10.1)	3 cortical lesions, HC lesions, 1 glioma	4L, 3R, 15 nonlateralised/generalised
Gascoigne et al. (2012)	Yes (7 days)	20	41	10.8 (2.5)	11.2 (2.6)	10M 10F	20M 21F	102.0 (10.6)	111.3 (11.2)	None	IGE
Giovagnoli et al. (1995)	No (13 days)	24	25	38 (11.82)	37.5 (10.88)	14M 14F	13M 12F	–	–	None	12R 16L
Helmstaedter et al. (1998)	Yes (1 week)	55	21	26.9	29.4	27M 28F	11M 10F	100 (11)	110 (12)	10 none, 14 HS, 16 tumours, 4 heterotopia, 11 other TL	27R 28L
Hoefeijzers et al. (2013)	Yes (1 week)	17	18	65.5 (8.8)	68.3 (8.8)	9M 8F	7M 11F	123.2 (11.4)	119.2 (16.0)	None	–
Martin et al. (1991)	Yes (1 day)	21	21	31 (7.5)	40 (11.4)	10M 11F	6M 15F	91.4 (9.9)	101 (10.1)	6 postop	8R 13L
Mameniskiene et al. (2006)	Yes (4 weeks)	70	59	33 (9.5)	31 (9.5)	29M 41F	19M 40F	–	–	11 TL lesion	–
Manes et al. (2005)	Yes (6 weeks)	7	7	57 (8.1)	64	6M 1F	–	115.3 (8.5)	110.5 (6.7)	None	–
Muhlert et al. (2010)	Yes (1 day)	11	11	68.6 (9.9)	66.0 (8.3)	11M 1F	1M 11F	122.7 (6.0)	119.6 (13.0)	None	–
Muhlert et al. (2011)	Yes (3 weeks)	28 (14 TLE, 14 IGE)	15	46.4 (11.0) TLE 31.6 (14.6) IGE	33.3 (15.4)	4M/10F TLE 6M/8F IGE	7M 8F	112.0 (13.5) TLE 113.1 (15.5) IGE	117.5 (12.2)	7 MTS, 1 right glioma, 1 right stroke, 1 right epidermoid cyst	7R 4L 3 uncertain
Narayanan et al. (2012)	Mixed (4 weeks)	14	17	33.6 (10.1)	37.4 (12.8)	6M 9F	3M 14F	98.3 (6.7)	101.6 (5.2)	5 left HS or atrophy, 3 right HS or atrophy	9L 6R
Tramoni et al. (2011)	Yes (6 weeks)	5	15	42.6 (9.3)	42.3 (9.6)	4M 1F	–	115 (10.4)	–	1 right MTS, 1 left hippocampal dysplasia	1L 2R 2 uncertain
Wilkinson et al. (2012)	Yes (6 weeks)	27	22	36.7	41.14 (12.24)	–	–	104.96	111.14 (10.10)	15 left HS 12 right HS	–

Studies reporting separate statistics for right and left TLE patients have been combined in this summary. M = male, F = female, – = information not presented, R = right, L = left, Bi = bilateral, HS = hippocampal sclerosis, postop = undergone epilepsy surgery, IGE = idiopathic generalised epilepsies. MTS = Mesial temporal sclerosis.

**Table 4:** Case reports of ALF in epilepsy: methodology evaluation.

Authors (year)	Matched controls?	Test material	Recall & recognition?	Ceiling & floor effects avoided?	Rehearsal avoided?	Immediate delay after 15 sec?	Matching procedure?	Initial learning equated?
Butler et al. (2012)	Age – yes IQ – yes	Verbal Visuo-spatial	Yes	No	No	No	No	Yes
Butler and Zeman (2008a)	Age – yes IQ – no	Verbal Visuo-spatial	No	No	No	No	Yes	Yes
Cronel-Ohayon et al. (2006)	Age – yes IQ – no	Verbal Visuo-spatial	No	Yes	No	No	No	Yes
Holdstock et al. (2002)	Age – yes IQ – yes	Verbal	No	No	Yes	Yes	Yes	Yes
Jansari et al. (2010)	Age – yes IQ – yes	Verbal	Yes	No	Yes	No	No	Yes
Kapur et al. (1996)	Age – yes IQ – no	Verbal Visuo-spatial	Yes	No	No	No	No	Yes
Kapur et al. (1997)	Age – yes IQ – yes	Verbal Visuo-spatial	Yes	No	No	No	No	Yes
Kemp et al. (2012)	Age – yes IQ – yes	Verbal Visuo-spatial	Yes	Yes	No	No	Yes	Yes
Lucchelli and Spinnler (1998)	Age – yes IQ – no	Verbal Visuo-spatial	No	No	No	No	No	Yes
Mayes et al. (2003)	Age – yes IQ – yes	Verbal Visuo-spatial	Yes	No	Yes	Yes	Yes	Yes
O'Connor et al. (1997)	Age – yes IQ – no	Verbal	No	No	No	No	Yes	Yes

**Table 5:** Group studies of ALF in epilepsy: methodology evaluation.

Authors (year)	Matched controls?	Test material	Recall & recognition?	Ceiling & floor effects avoided?	Rehearsal avoided?	Immediate delay after 15 sec?	Matching procedure included?	Initial learning equated?
Bell et al. (2005)	Age – yes IQ – no	Verbal Visuo-spatial	No	Yes	Yes	No	Yes	No
Bell (2006)	Age – yes IQ – no	Verbal	Yes	Yes	Yes	No	No	No
Blake et al. (2000)	Age – yes IQ – yes	Verbal	Yes	No	Yes	No	Yes	Yes
Butler et al. (2007)	Age – yes IQ – yes	Verbal Visuo-spatial	Yes	No	Yes	No	Yes	Yes
Butler et al. (2009)	Age – yes IQ – yes	Verbal Visuo-spatial	No	Yes	No	No	Yes	Yes
Butler et al. (2013)	Age – yes IQ – yes	Verbal Visuo-spatial	No	Yes	No	No	Yes	Yes
Davidson et al. (2007)	Age – yes IQ – yes	Verbal Visuo-spatial	Yes	Yes	Yes	No	Yes	No
Evans et al. (2013)	Age – yes IQ – yes	Verbal Visuo-spatial	Yes	Yes	Yes	Yes	Yes	6/8 tests
Fitzgerald, Mohamed, et al. (2013) and Fitzgerald, Thayer, et al. (2013)	Age–yes IQ – yes	Verbal Visuo-spatial	No	No	No	No	Yes	Yes
Gascoigne et al. (2012)	Age – yes IQ – no	Verbal	Yes	No	No	Yes	Yes	Yes
Giovagnoli et al. (1995)	Age – yes IQ – no	Visual	No	Yes	No	No	Yes	No
Helmstaedter et al. (1998)	Age – yes IQ – no	Verbal Visuo-spatial	No	Yes	Yes	No	No	No
Hoefeijzers et al. (2013)	Age – yes IQ – yes	Verbal	Yes	Yes	Yes	No	Yes	Yes
Martin et al. (1991)	Age – no IQ – no	Verbal	No	Yes	Yes	No	Yes	Yes
Mameniskiene et al. (2006)	Age – yes IQ – no	Verbal Visuo-spatial	No	Yes	No	No	No	No
Manes et al. (2005)	Age – yes IQ – yes	Verbal Visuo-spatial	Yes	No	No	No	No	Yes
Muhlert et al. (2010)	Age – yes IQ – yes	Verbal	No	Yes	No	Yes	Yes	Yes
Muhlert et al. (2011)	Age – yes^a^ IQ – yes^a^	Verbal Visuo-spatial	Yes	Yes	Yes	Yes	Yes	Yes
Narayanan et al. (2012)	Age – yes IQ – yes	Verbal Visuo-spatial	Yes	No	No	No	Yes	Yes
Tramoni et al. (2011)	Age – yes IQ – yes	Verbal Visuo-spatial	Yes	No	No	No	No	Yes
Wilkinson et al. (2012)	Age – yes IQ – yes	Verbal Visuo-spatial	No	Yes	No	Yes	Yes	Yes

a A subset of control participants were examined with matched age and IQ to the TLE patients.
